# Genome-Wide Identification and Functional Characterization of β-Agarases in *Vibrio astriarenae* Strain HN897

**DOI:** 10.3389/fmicb.2020.01404

**Published:** 2020-06-24

**Authors:** Yupeng Liu, Xingkun Jin, Chao Wu, Xinyuan Zhu, Min Liu, Douglas R. Call, Zhe Zhao

**Affiliations:** ^1^Department of Marine Biology, College of Oceanography, Hohai University, Nanjing, China; ^2^Paul G Allen School for Global Animal Health, Washington State University, Pullman, WA, United States

**Keywords:** agarolytic activity, β-agarase, gene knockout, glycoside hydrolase (GH), *Vibrio astriarenae*, whole genome sequencing

## Abstract

The genus *Vibrio* is a genetically and metabolically versatile group of heterotrophic bacteria that are important contributors to carbon cycling within marine and estuarine ecosystems. HN897, a *Vibrio* strain isolated from the coastal seawater of South China, was shown to be agarolytic and capable of catabolizing D-galactose. Herein, we used Illumina and PacBio sequencing to assemble the whole genome sequence for the strain HN897, which was comprised of two circular chromosomes (Vas1 and Vas2). Genome-wide phylogenetic analysis with 140 other *Vibrio* sequences firmly placed the strain HN897 into the Marisflavi clade, with *Vibrio astriarenae* strain C7 being the closest relative. Of all types of carbohydrate-active enzyme classes, glycoside hydrolases (GH) were the most common in the HN897 genome. These included eight GHs identified as putative β-agarases belonging to GH16 and GH50 families in equal proportions. Synteny analysis showed that GH16 and GH50 genes were tandemly arrayed on two different chromosomes consistent with gene duplication. Gene knockout and complementation studies and phenotypic assays confirmed that *Vas1_1339*, a GH16_16 subfamily gene, exhibits an agarolytic phenotype of the strain. Collectively, these findings explained the agar-decomposing of strain HN897, but also provided valuable resources to gain more detailed insights into the evolution and physiological capability of the strain HN897, which was a presumptive member of the species *V. astriarenae*.

## Introduction

Agar, the major ingredient of the cell wall in many red algae, is a complex water-soluble polysaccharide that is composed of agarose and agaropectin (Renn, 1997). Agarose is a linear macromolecule composed of repeated D-galactose and 3,6-anhydro-L-galactose units joined by α-1,3- and β-1,4-glycosidic bonds, while agaropectin is a derivative of agarose with repeated units of methylated, pyruvated, sulfonated, or glycosylated galactose moieties (Lee et al., 2017). Agarase is an important agarolytic enzyme involved in producing oligosaccharides or monomeric sugars from agarose, functions that are of considerable interest to the food, cosmetics and medical industries (Fu and Kim, 2010; Yun et al., 2017).

β-agarases (EC 3.2.1.81) are the most common class of agarase and to date, more than 30 have been functionally verified and documented in the carbohydrate-active enzymes (CAZymes) database (Chi et al., 2012). Based on amino acid sequence similarity, all β-agarases can be classified into the glycoside hydrolase (GH) subfamilies, including GH16, GH50, GH86, and GH118 (Lee et al., 2012). The mechanism of these enzymes is well established (Ekborg et al., 2006; Fu and Kim, 2010; Pluvinage et al., 2013; Yun et al., 2017). Initially, the endo-type β-agarase, including GH16 and GH86, hydrolyze internal β-1,4 glycosidic linkages of agarose and release a series of neoagarooligosaccharides (NAOS) (Ohta et al., 2004; Hehemann et al., 2012; Chi et al., 2014; Naretto et al., 2019). Other endo-type β-agarases, including GH50 and GH118, act in cleaving the terminal β-1,4 linkages at the non-reducing end of NAOS, and ultimately release hydrolysis products such as neoagarobiose (NeoDP2) (Xie et al., 2013; Yu et al., 2020).

The majority of agarase-producing bacteria are from marine environments including taxonomically distinct genus such as *Pseudomonas* (Kang et al., 2003; Hsu et al., 2015), *Alteromonas* (Chi et al., 2014), *Microbulbifer* (Ohta et al., 2004; Jonnadula and Ghadi, 2011), and *Agarivorans* (Fu et al., 2009; Lin et al., 2012). Although agar utilization is not particularly common in *Vibrio* species, several β*-*agarases have been identified (Lee et al., 2014; Yun et al., 2015; Yu et al., 2020). For example, agarases have been purified and characterized from different agarolytic *Vibrio* strains (Aoki et al., 1990; Sugano et al., 1993; Araki et al., 1998), and a set of β*-*agarase-encoding genes have been described and functionally validated as recombinant proteins (Sugano et al., 1993; Dong et al., 2006; Zhang and Sun, 2007; Liao et al., 2011). The agarolytic system of the *Vibrio* sp. strain EJY3 has been studied extensively and this strain is the only agarolytic *Vibrio* that harbors four GH50 β-agarases (Lee et al., 2014; Yun et al., 2015; Yu et al., 2020).

Vibrios are Gram-negative, obligate heterotrophic bacteria that inhabit marine and estuarine environments as free-living populations or in association with a variety of aquatic organisms (Thompson et al., 2004). This is a diverse genus with more than 120 validated species (Sawabe et al., 2013; Parte, 2018). Some *Vibrio* species are pathogenic to marine animals and people, while the majority are mostly non-pathogenic and are found as the normal part of marine microbial communities (Thompson and Polz, 2006). *Vibrio* species are known to use complex organic carbohydrates, such as polysaccharides (cellulose, chitin, and agar etc.), as carbon and energy sources and play a significant role in environmental carbon cycling (Hunt et al., 2008; Zhang X. H. et al., 2018).

A marine *Vibrio* (strain HN897) was recently isolated from the coastal seawater of South China and shown to be agarolytic and able to catabolize D-galactose (Zhang J. Y. et al., 2018). In this study, we have generated a high-quality genome assembly of the strain HN897 and described new agarase genes, including functional validation. Results from this work further our understanding of agar-decomposing organisms and provide a valuable resource to gain more detailed insights into the evolution and physiological capability of agar-degrading organisms that play a vital role in carbon cycling.

## Materials and Methods

### Bacterial Strains, Plasmids and Growth Conditions

The bacterial strains and plasmids used in this study are listed in the Supplementary Table S1. The strain HN897 was isolated from seawater in the South China coastal areas. In brief, thiosulfate citrate bile salts sucrose (TCBS) plates were used to isolate 150 putative *Vibrio* strains, of which three were phenotypically tested for agarose-degrading activity. The most active strain was designated HN897. All the *Vibrio* strains were routinely grown in trypticase soy broth (TSB; BD) supplemented with 1% (w/v) NaCl or on TSB agar plates (TSA) supplemented with 1% (w/v) NaCl at 30°C. *Escherichia coli* S17 λpir was used in gene deletion experiments and was cultured in Luria-Bertani (LB; BD) medium. Suicide plasmid pDM4 was used to generate gene knockouts and expression vector pMMB207 was used for complementation experiment (Morales et al., 1991; Milton et al., 1996). Unless otherwise indicated, chloramphenicol was used at the concentration of 34 μg/mL in the media.

### Genomic DNA Isolation and Whole Genome Sequencing

Genomic DNA was extracted from the strain HN897 using the CTAB method with minor modification (Liu et al., 2017). The concentration, quality and integrity of the DNA samples were evaluated using a NanoDrop 2000 Spectrophotometer (Thermo Scientific, United States) and a Bioanalyzer 2100 (Agilent Technologies, United States), respectively. Insertion libraries [400 bp pair-end (PE250)] were constructed with Illumina TruSeq sample preparation reagents according to manufacturer instructions and sequenced using an Illumina MiSeq (Illumina, United States) instrument. Larger 20k bp insertion (S20K) libraries were prepared by SMRTbell Template Prep Kit 1.0 according to the PacBio protocol, and sequenced using a PacBio RS II platform (Pacific Biosciences, United States).

### Genome Assembly and Annotation

For the Illumina short reads, the adaptor sequences were removed by Adapter Removal v2.1.7 (Mikkel et al., 2016), and the k-mers for the paired-end libraries were tallied using SOAPec v2.0 (Luo et al., 2012) and verified using SPAdes v3.9.0 (Bankevich et al., 2012) followed by assembly with A5-miseq v20150522 software (Tritt et al., 2012). The PacBio sequences were assembled using HGAP4 (Chin et al., 2016) and CANU v1.6 (Koren et al., 2017). Subsequently, the assembled contigs from both Illumina and PacBio reads were merged, and gaps were filled using MUMmer v3 (Delcher et al., 1999). Finally, the full genome assembly was generated with Pilon v1.22 (Walker et al., 2014) software. Microbial genes were predicted with GeneMark.hmm v4.32 using the Heuristic models option (Besemer et al., 2001). The coding sequences (CDS) of the putative genes were aligned to NCBI-NR database using BLASTP to retrieve annotations with an E-value cutoff of 1e-6. Clusters of orthologous genes (COGs) were annotated with eggNOG-mapper (evolutionary genealogy of genes: non-supervised orthologous groups) with eggNOG database v4.5 and an *E*-value cutoff of 1e-6 (Huerta-Cepas et al., 2017). The final assembled circular chromosomes (CGs) were compared to closely related species by sequence alignment using tBLASTx with the following thresholds: *E*-value: 1e-10; identity: 90%; alignment length: 100. CG maps were constructed and visualized based on the sequence alignment blocks using GView Server (Stothard et al., 2019). KAAS (KEGG Automatic Annotation Server)^1^ was used to assign the KEGG (Kyoto Encyclopedia of Genes and Genomes) orthology annotations^2^ for the predicted proteins. Proteins were functionally classified via subfamily domain modules that had been annotated using PfamScan v1.6, with Pfam32.0 database (Finn et al., 2016). Putative CAZymes profiles were identified using Hmmscan v3.1b2 (Krogh et al., 1994).

### Genome-Based Taxonomic Classification

Using the representative genomes in Genome Taxonomy Database (GTDB Release 89) (Parks et al., 2020) as references, the genome-based taxonomy of HN897 was classified using GTDB-tk ‘classify_wf’ (Chaumeil et al., 2019). Maximum-likelihood ML (WAG + gamma model) trees were constructed using FastTree2 (Price et al., 2010) based on multiple sequences alignment of 120 concatenated bacterial single-copy marker protein sequences according to the GTDB genome phylogeny (Parks et al., 2018). Further, the quality of HN897 genome assembly was assessed based on lineage-specific phylogenetic markers using CheckM (Parks et al., 2015). A precomputed and visualized genome-wide phylogeny with taxonomic annotations showing the members in γ-*proteobacterial* order was downloaded from AnnoTree (Mendler et al., 2019). As a complementary analysis, a ML phylogenetic tree, based on 16S rDNA sequences, was constructed using FastTree2 with a GTR + gamma model and MAFFT-aligned sequences from Al-Saari et al. (2015). This latter analysis included other *Vibrio* data having a high sequence similarity (>98%) to HN897. All the ML trees were constructed and visualized by iTOL (Letunic and Bork, 2019).

### Sequence Clustering and Synteny Analysis of β-Agarase-Coding Genes

Based on the CAZymes classifications, the predicted full-length proteins of GH subfamilies were abstracted and characterized for conserved enzymatic domains (excluding carbohydrate-binding CBMs and lectins) were identified using InterProScan (Jones et al., 2014). Identified GH domain sequences were aligned using COBALT (Constraint-based Multiple Alignment Tool) (Papadopoulos and Agarwala, 2007) and an unrooted ML tree was constructed using FastTree2 with the WAG + Gamma models. The protein domain architectures for putative β-agarases were further inspected and visualized according to SMART database (Letunic and Bork, 2018). The structure-based sequence similarity comparisons were based on alignment by MUSCLE v3.8.31, and the secondary structure information was parsed and annotated using ESPript 3.0 (Robert and Gouet, 2014).

A total of 45 subject genome assemblies were used for synteny comparisons with the strain HN897. These assemblies were selected because they (i) included genes that have high sequence similarities to the β-agarase from HN897 (>60% amino acid identity); (ii) appeared phylogenetically related to HN897 according to genome-based taxonomic classification from the present study and the phylogeny inference of the strain C7 (Al-Saari et al., 2015). A total of 46 assemblies were used and the genomic loci spanning eight β-agarases from HN897 were inspected and compared using MultiGeneBLAST v1.1.14 (Medema et al., 2013).

### Construction of Deletion Mutants and Complementation

All deletion mutants were constructed by allelic exchange as described previously (Zhao et al., 2018). Briefly, deletion cassettes for chromosomal in-frame deletions were generated through the splice-overlap-extension (SOE) method (primers listed in Supplementary Table S2), which joins two approximately 400-bp amplicons corresponding to genomic regions flanking β-agarase genes. The deletion cassettes were then cloned into a suicide plasmid (pDM4) using standard cloning procedures. Each resulting construct was transformed into chemically competent cells of *E. coli* S17-1 λpir, and then transferred into HN897 by conjugation. Mutant strains were selected on TCBS plates containing chloramphenicol (6.8 μg/mL) followed by a 10% (w/v) sucrose selection process. Each positive deletion mutant was experimentally confirmed using PCR with gene-specific primers spanning the deleted region.

For complementation tests, the complete ORF of each indicated target gene was amplified using gene-specific primers (Supplementary Table S2) and cloned into the vector pMMB207. The insertion success of constructed plasmid was checked by Sanger sequencing, and only the positive ones were introduced into the mutant strain by conjugation.

### Assays of Agarolytic and Agarase Activity

An agar-spot assay was used to evaluate the agarolytic activity of each HN897 strain. Briefly, each indicated strain was incubated overnight and spotted (5 μl) onto a fresh TSA plate supplemented with 1% (w/v) NaCl and 0.5 mM IPTG. For the complementation strain, overnight culture was diluted 1:100 into TSB medium supplemented with chloramphenicol and 1% (w/v) NaCl and was cultured with shaking at 30°C. When the OD_600_ reached ∼0.6, IPTG (1 mM final) was added to induce protein expression and then continued to culture for 5 h. The induced culture was subjected to agar-spot assay as described above. The plates were incubated at 30°C for 12 h, and the colonies that formed transparent lysis zones on agar plates were classified as agarolytic.

Agarase activity of each indicated strain was measured using the 3, 5-dinitrosalicylic acid (DNS) method as described previously (Miller, 1959). Briefly, after 24 h culture bacterial cells were pelleted by centrifugation (14000 *g*, 5 min) at 4°C and resulting supernatant was collected and kept for the agarose activity assay. For the complementation strain, the bacterial cells were cultured with chloramphenicol and IPTG as aforementioned. Culture supernatant (1 mL) was mixed with 1 mL 10 mM sodium citrate buffer (pH = 5) containing 0.2% agarose that was pre-solubilized using a conventional microwave oven. After incubation at 30°C for 60 min, 750 μL of DNS solution (36.4 g of potassium sodium tartrate, 1.2 g of DNS, and 83.2 mL 5% NaOH in 200 mL distilled water) was added to the cultures and boiled at 100°C for 10 min, followed by ice-cold water-based cooling for 2 min. The absorbance at 520 nm for each sample was measured using spectrophotometer. Distilled water (1 mL) was included as a blank control. For the agarase activity assays, the extent of agarose reduction was estimated using a standard curve of D-galactose (Sangon, Shanghai, China).

## Results

### High-Quality Genome Sequence of Marine *Vibrio* Strain HN897

Contig assembly were generated using 4,713,440 (96.86% of total) Illumina paired-end reads with 233 × coverage. Scaffolds were generated using 152 × coverage of PacBio sequences (11,636-bp N_50_). The contigs generated by both short- and long-reads were scaffolded to fill gaps and increased the accuracy of sequence consensus of assembly. The complete genome for HN897 included two circular chromosomes spanning 3,120,326 bp with 46.06% GC, and 1,679,007 bp with 44.82% GC, respectively (Supplementary Figures S1, S2). The larger chromosome (“Vas1”) encompasses 2,801 coding sequences (CDSs), 110 tRNAs and 34 rRNAs while the smaller chromosome (“Vas2”) consists of 1,487 CDSs, 6 tRNA genes, and 16 miscellaneous ncRNAs (Supplementary Table S3).

The BLAST-NR based annotations allocated 2,736 and 1,420 gene hits for Vas1 and Vas2, respectively, and approximately half of the hits were derived from a single genome (*Vibrio* sp. C7) with >90% of identities and alignment coverage on average (Supplementary Figure S3). For the remaining top 10 hit species, the genus *Vibrio* was predominant although *Photobacterium gaetbulicola* was also identified. *P. gaebulicola* is a member of *Vibrionaceae* (Supplementary Figure S3). The CheckM assessment and classification showed the genome assembly of HN897 was 98.21% complete and 3.24% contaminated, ensuring a critical foundation for further biological inference of this particular strain. It is worth mentioning that the genome contaminations reported by CheckM was based on occurrence of duplications of single-copy marker genes, which might be true expanded paralogs in HN897 genome. The full standard metrics for HN897 assembly according to the Genomic Standards Consortium (GSC) (Bowers et al., 2017) are shown in Supplementary Table S3.

### Taxonomic Assignment of HN897 Genome

**FIGURE 1:**
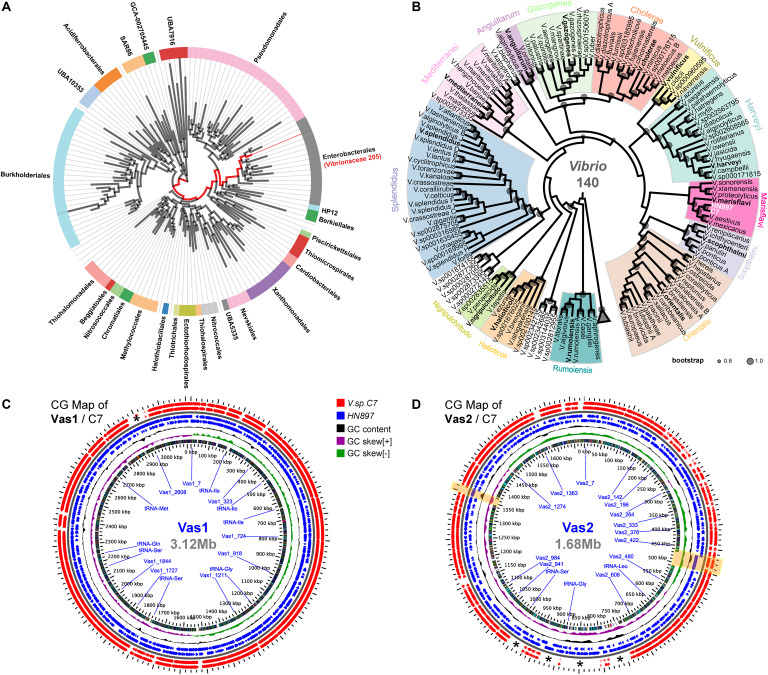
Phylogenetic relationships and genomic comparisons of HN897 with other *Vibrio* strains. The genome taxonomy of HN897 was classified by GTDB-tk ‘classify_wf’ comparing to representative genomes in GTDB (Chaumeil et al., 2019). Maximum-likelihood (WAG + gamma model) species trees were estimated by FastTree2 based on multiple sequences alignment of 120 concatenated bacterial single-copy marker protein sequences according to the GTDB genome phylogeny (release89) (Parks et al., 2020). **(A)** A monophyletic branch formed by a total of 205 members (red branch) of the γ-*proteobacterial* order *Vibrionaceae* according to AnnoTree (Mendler et al., 2019). **(B)** The genome of HN897 was placed onto aforementioned *Vibrionaceae* lineage, of which only the 140 members of genus *Vibrio* were shown (see Supplementary Figure S4 for complete tree). *Vibrio* clades were defined according to Sawabe et al. (2013). The sized circles in each node branch denoted the robustness of internal branch placement assessed using bootstrap support (0.8∼1.0). Triangle denoted the 65 collapsed non-*Vibrio*s. Two circular genome (CG) maps generated with the GView Server show the comparisons of Vas1 **(C)** and Vas2 **(D)**, respectively, with closely related *V. astriarenae* C7 genome using tBLASTx (threshold: *E*-value of 1e-10; identity of 90%; alignment length of 100). For both maps, the 2x zoomed view depicting the contents of the feature rings (starting with the outermost ring) are as follows: Rings 1,2: negative and positive stranded CDSs from strain C7; Rings 3,4: negative and positive stranded CDS from strain HN897; Ring 5 shows GC content; Ring 6 shows GC skew and Ring 7 shows COG functional categories (see Supplementary Figures S1, S2 and Supplementary Table S4 for details). Asterisks (*) marks the regions of genomes that lack pair-wise sequence alignments. Two genome regions shaded by light yellow boxes show the loci of predominant long tandem repeats on Vas2.

According to GTDB, all 205 representative species of *Vibrionaceae* formed a monophyletic family level group (red clade in Figure 1A). Notably, the parent taxon of *Vibrionaceae* was *Vibrionales* under the NCBI taxonomy, but reclassified as *Enterobacterales* by GTDB taxonomy after taxonomic-rank normalization (Parks et al., 2018). Further, the genome of HN897 was firmly placed into the aforementioned *Vibrionaceae* subtree, together with 140 *Vibrio* members, and more precisely within a subclade shared with *V. astriarenae* strain C7 (Figure 1B and Supplementary Figure S4). Alignment-based classification showed that the average nucleotide identity (ANI) between HN897 and C7 was 96.6% (Supplementary Table S3), further indicating the close relatedness between these two strains. We further constructed two CG maps comparing the sequences of Vas1 and Vas2 with that of contig-level genome assembly of C7 respectively. Using BLAST comparisons conducted between CDS translations from both strains, the detected regions with high sequence similarity (90% identity threshold) included most of the coding regions in each chromosome (95.5% CDSs for Vas1; 77.2% for Vas2). The positions of contig boundaries and potential fragmentations for C7 assembly were revealed as marked with asterisks and shaded boxes shown in Figures 1C,D.

### Interpretation of the HN897 Genome via Functional Annotations

**FIGURE 2:**
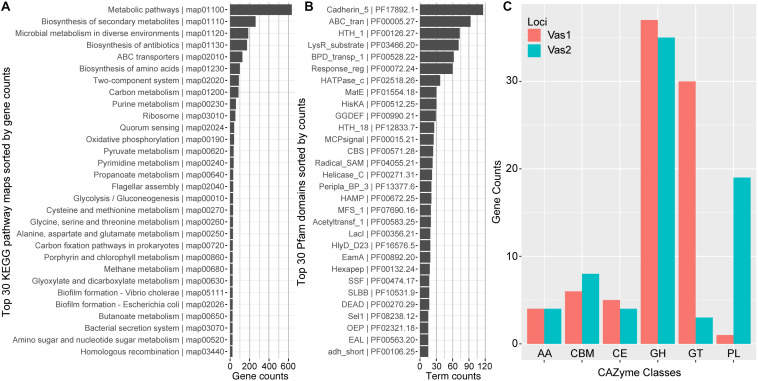
Functional network modules and characterized protein domains encoded by HN897 genome. Bar plots showed the numbers of **(A)** genes that associate with each indicated KEGG pathway maps; **(B)** indicated Pfam protein domain models contained in the genome; and **(C)** genes that were classified into the same indicated CAZyme class. Both the KEGG pathway map entries and Pfam terms were labeled with accession numbers as the suffix. AA, auxiliary activity; CBM, carbohydrate-binding module; CE, carbohydrate esterase; GH, glycoside hydrolase; GT, glycosyl transferase; PL, polysaccharide lyase.

In total, 2,344 (54.66%) predicted CDSs were placed into ortholog groups according to the KEGG Orthology (KO) database. Most of the KO-annotated genes were related to metabolic pathways (634), especially anabolism such as biosynthesis of secondary metabolites (262), antibiotics (174) and amino acids (99). In addition, many other KO-annotated genes were related to different cellular signaling pathways, including ABC transporters pathway (126), two-component systems (99) and quorum sensing (41). Importantly, a set of gene components that take part in carbon (86) and pyruvate (36) metabolism, and glycolysis/gluconeogenesis (30) appeared relatively complete (Figure 2A and Supplementary Table S4).

The majority of CDSs for Vas1 (87.9%) and Vas2 (81.4%) had at least one Pfam model hit. The most abundant domain was cadherin_5 (PF17892) with 116 hits (Figure 2B), amongst which 111 were tandemly arranged within the longest CDS, Vas2_433 (12,014 aa, WP_164651137.1) (The larger shaded box in Figure 1D). Notably, the second longest CDS, Vas_1198 (5,779 aa, WP_164650763.1) also contained a single cadherin_5 domain with an additional 19 N-terminal tandem repeats of t1ss_rpt_143 domain (tigr:TIGR03660), and a C-terminal domain cluster including one each of tandem-95 repeat (CDD: NF012211), hemolysinCabind (PF00353) and peptidase_M10_C (PF08548) (the smaller shaded box in Figure 1D). Consistent with the KEGG annotation results, the ABC_transporter (PF00005) domains were abundant, which was not uncommon as this group forms the largest protein family in many other sequenced bacterial genomes (Saurin et al., 1999). Many signal transduction related domains were found with high frequency, such as methyl-accepting chemotaxis protein (MCP) domain (MCPsignal, PF00015), HAMP (PF00672), response regulator receiver domain (Response_reg, PF00072), and his kinase A (HisKA, PF00512). Notably, the latter two domains are critical components of two-component system regulators. The other abundant domains included evolutionarily conserved helix-turn-helix DNA binding motifs, such as HTH1 (PF00126), HTH18 (PF12833), and LacI (PF00356) that regulates gene expression (Figure 2B).

Most of the agarolytic bacteria encoded a large repertoire of putative CAZymes and carbohydrate-binding proteins (Barbeyron et al., 2016; Osterholz et al., 2016). HN897 had a total of 72 glycoside hydrolases (GHs), 33 glycosyl transferases (GTs), 20 polysaccharide lyases (PLs), 9 carbohydrate esterases (CEs), and 8 auxiliary activities (AAs). Carbohydrate-binding modules (CBMs) were also identified (Figure 2C and Supplementary Table S4).

### The β-Agarase Gene Repertoire of Strain HN897

**FIGURE 3:**
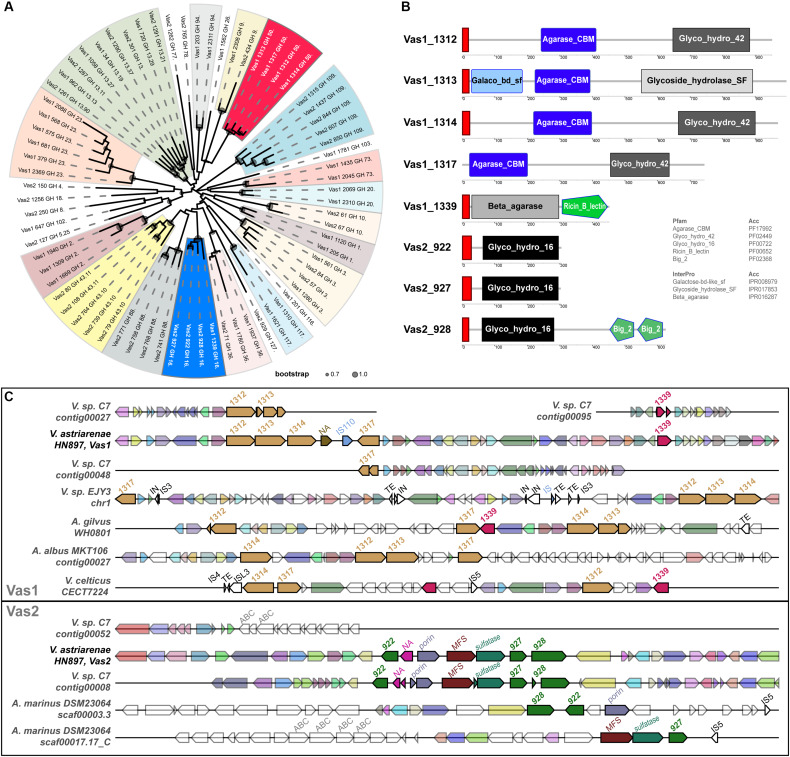
Comprehensive comparisons of β-agarases at the sequence similarity, domain architecture and synteny level. **(A)** A maximum-likelihood (WAG + gamma model) tree was estimated by FastTree2 based on the multiple sequence alignments of the InterProScan captured enzymatic protein domains of a total of 72 GHs (see section “Materials and Methods” and Supplementary Table S5 for details). Each branch was annotated with designated gene name suffixed with GH subfamily number. Clades containing at least two members from the same GH subfamily were shaded, and the ones for GH16 and GH50 were marked by blue and red, respectively. The circles within each branch junctions were sized proportionally according to bootstrap support (0.7∼1.0). **(B)** The schematic diagram depicted the total domain structure of the GH16 and GH50 family members. Each scaled box denoted a functional module labeled with term shortname, and of which the label color denoted database source (white: Pfam; black: InterPro). The red boxes denoted signal peptides. **(C)** Syntenic gene tracks showed the β-agarases-containing loci on both Vas1 and Vas2 (as queries), respectively, comparing to genomic loci in each indicated genome (as subjects). The colors of the gene arrows represented BLAST identities across both intra- and inter-specific comparisons. The white gene arrows denoted flanking genes without BLAST hits to the query. The size-scale and color tone for each gene block was only comparable within its own subpanel. The mobile elements including insertion sequence (IS), integrase (IN) and transposable element (TE) with transposase were also labeled on the top of each gene arrow.

Eight GH subfamily members were similar to β-agarases, including all the members of GH16 and GH50 in HN897 (Supplementary Table S5). As expected, a ML tree based on conserved enzymatic domains from 72 GH protein sequences revealed congruence at the GH subfamily level for all the GHs. The eight β-agarases from HN897 were included in subclade of GH50 and GH16 (Figure 3A).

The identified N-terminal hydrophobic signal peptides showed that members of both GH50s and GH16s were likely to be secretory, with the only exception of Vas1_1317 (WP_164648142.1) that lacked such structure (Figure 3B). As expected, the domains of agarase_CBM (PF17992) and glyco_hydro_42 (PF02449) were only shared by the GH50 members while the glyco_hydro_16 domain (PF00722) was specific to GH16 members. Vas1_1313 (a GH50 member, WP_164648138.1) harbored an alternative glycoside_hydrolase_SF domain (IPR017853) in its C-terminus, and an extra galactose-bd-like_sf domain (IPR008979) in its N-terminus. Vas1_1339 (a GH16 member, WP_164648163.1) possessed an alternative domain, namely beta_agarase (IPR016287) in N-terminal region, and an extra ricin_B_lectin domain (PF00652) in its C-terminal region. Vas2_928 (a GH16 member, WP_164650524.1) had two extra tandemly repeated domains namely bacterial_Ig-like_2 (PF02368) in its C-terminal end.

Gene clusters for regions spanning the eight β-agarase genes of HN897 were compared with 45 bacterial genomes (Supplementary Table S5). The intraspecific pairwise sequence identity for the GH50s from Vas1 ranged between 34% and 48%, and ranged between 37 and 66% for GH16s from Vas2. The four Vas1 GH50-encoding genes were arranged tandemly, with the latter two separated by a non-annotated putative gene followed by an IS110 transposase (upper panel of Figure 3C). A single GH16 gene, *Vas1_1339*, was located downstream of the above mentioned GH50s cluster, with an interval of 22 protein-coding genes. On Vas2, the three GH16s were also arranged tandemly with the upstream two separated by four non-GH family genes (bottom panel of Figure 3C). As expected, the overall synteny and collinearity between HN897 and C7 were consistent for both Vas1 and Vas2, except for the absence of one GH50 gene, Vas1_1314 (WP_164648139.1), and prevalent frameshifts in several other GH subfamily genes on C7 assembly (Figure 3C and Supplementary Table S5). Moreover, the one-to-one sequence similarities of β-agarases encoding genes was >99% between these two strains (Supplementary Table S5), implying recent ancestry or conserved function. The quadripartite tandem array of GH50s found on Vas1 were only partially evident in other species including *Vibrio sp.* EJY3 (*Vibrio natriegens* according to GTDB), *Agarivorans gilvus* strain WH0801, *Agarivorans albus* MKT106 and *Vibrio celticus* strain CECT 7224. Notably, the cross-species sequence similarities of encoded GH50 protein sequences between HN897 and EJY3 were high (72∼92%). Nevertheless, genome-wide synteny was less consistent as evidenced by various degrees of local translocations and inversions (upper panel of Figure 3C). Comparatively, the tripartite tandem array of GH16 on Vas2 was only found in two separate contigs of *Aliagarivorans marinus* DSM 23064, but with certain rearrangement (bottom panel of Figure 3C).

### Functional Verification of the Vas1_*1339*-Agarolytic Phenotype

**FIGURE 4:**
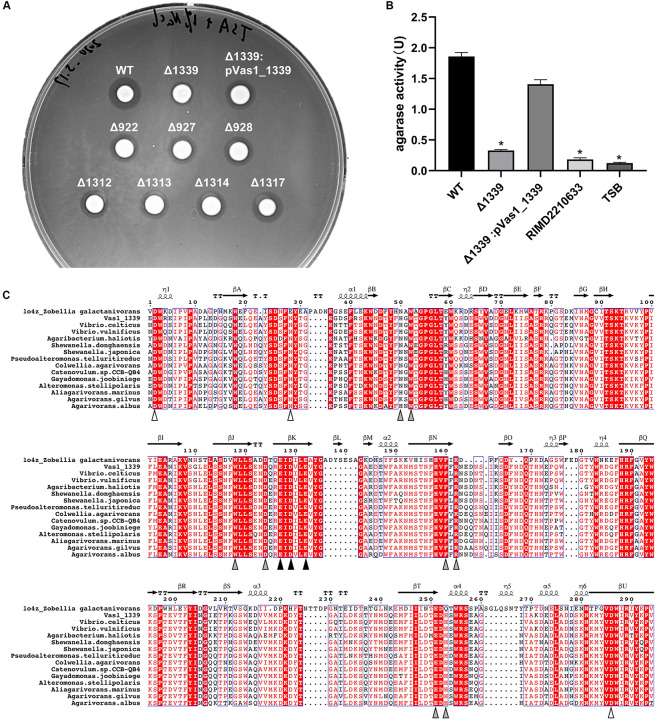
A GH16_16-encoding gene *Vas1_1339* is indispensable for the agarolytic phenotype of HN897. **(A)** Results of agar-spot assays comparing the wild type (WT), deletion mutants and complementation strain. Each strain was spotted on a solid medium containing agar as substrate. The colonies that formed transparent lysis zones on agar plates were classified as agarolytic. **(B)** Agarase activity of cell-free culture supernatants was measured as described in section “Materials and Methods” from the wild type (WT), Δ*1339* mutant and Δ*1339*:p*Vas1_1339* strains. The blank TSB medium and the culture supernatant of *V. parahaemolyticus* strain RIMD2210633 were included as controls. The data were expressed as means ± SEM of agarase activity (U/mL) from three independent experiments. Statistical analysis was performed with one-way ANOVA and *P* < 0.05 were considered statistically significant. Pairwise comparisons were conducted using a Tukey–Kramer multiple comparisons test and the analyses were conducted using the SPSS software. **(C)** Multiple sequence alignment of the GH16 domain comparing Vas1_1339 to other orthologous β-agarases (see Supplementary Table S7 for details). The secondary structure information was based on resolved 3D structure of ZgAgaB (Protein Data Bank: 1o4z) from *Zobellia galactanivorans* (Naretto et al., 2019). η-(3_10_) and α-Helices were shown as helices; β-Strands were shown as arrows; and β-Turns were labeled with TT. The color-filled pointing up triangles indicated the calcium binding sites (white), active sites (gray) and catalytic residues (black), respectively. The figure was drawn by ESPript 3.0 (Robert and Gouet, 2014). See Supplementary Figure S6 for the full-length sequence alignment.

We individually constructed knock-out (KO) strains for all the eight putative β*-*agarase genes found in HN897. Agar-spot assays showed that the deletion mutants for seven of these strains resemble the wild-type strain (Figure 4A). In contrast, the agarolytic activity was abrogated in the Vas1_*1339* deletion strain (Δ*1339*). *In-trans* expression of Vas1_*1339* in the knockout strain restored the agarolytic phenotype (Figure 4A). Enzymatic activity assays further showed that the agarase activity in culture supernatant of Δ*1339* strain (0.327 U/mL) was significantly reduced comparing to the wild-type strain (1.859 U/mL) and complemented strain (1.408 U/mL) (Figure 4B). As control, the blank TSB medium and the culture supernatant of *Vibrio parahaemolyticu*s strain RIMD2210633 (clinical isolate O3:K6) (Makino et al., 2003) both exhibited little to no agarase activity (Figure 4B).

Using PaperBLAST (Price and Arkin, 2017), we found that Vas1_*1339* was most similar to a β-agarase gene (ABL06969.1), *agaV*, from *Vibrio* sp. V134 (similarity: 99%, coverage: 100%) and differing by only two amino acid residuals (Leu^259^ and Pro^347^ in Vas1_*1339*, Ile^259^ and Leu^347^ in AgaV). The 16S rRNA based ML tree also suggested close relatedness between identified *V. astriarenae* strains (C7, C20, and presumptively, HN897) and strain V134, indicating that the latter was likely a strain of *V. astriarenae* (Supplementary Figure S5 and Supplementary Table S6).

A structure-referenced sequence alignment suggested that the underlying molecular basis for agarolysis was evolutionary conserved, at least within the β-agarase domain (IPR016287) of the sequences that we investigated (Supplementary Figure S6 and Supplementary Table S7). For instance, the active sites for enzymatic reactions were nearly invariant, particularly the catalytic residues that consisted of a tripartite motif “ExDxxE” (marked by black triangles in Figure 4C). Likewise, three separate residues on domain termini were highly conserved. These residues were known to function together as a cation-binding site (usually occupied by Ca^2+^, Naretto et al., 2019).

## Discussion

Earlier work demonstrated that the marine *Vibrio* isolate HN897 is capable of catabolizing agar as a sole carbon source and it exhibits agarose-degrading activity (Zhang J. Y. et al., 2018). Using sequence data from Illumina and PacBio platforms and other analytic advances and databases such as genome-based taxonomy and quantitative species definition (see Parks et al., 2015, 2018, 2020; Chaumeil et al., 2019), we were able to determine with a high degree of confidence that the strain HN897 belonged to the *Vibrio astriarenae* (Figure 1B). This conclusion was further supported by high matching scores for sequence alignments (Supplementary Table S3), and by analysis of 16S rDNA sequences (Zhang J. Y. et al., 2018; Supplementary Figure S5).

The maximum likelihood analysis placed both HN897 and *Vibrio astriarenae* C7 within the Marisflavi clade of the *Vibrio* genus (Figure 1B). This clade was originally defined as a group of three species, *Vibrio aestivus*, *Vibrio marisflavi* and *Vibrio stylophorae* (Sheu et al., 2011) based on the 16S rDNA comparisons. Subsequent studies have added a new species, *Vibrio mexicanus*, together with *V. aestivus* to form a monophyletic group with *V. marisflavi* that is well separated from *V. stylophorae* (González-Castillo et al., 2015). In contrast, analysis based on multilocus sequence typing suggests that the *V. mexicanus*–*V. aestivus* cluster is well separated from *V. marisflavi*.

While different phylogenetic conclusions may be evident from different methodologies, we submit that genome-level comparisons are likely to be more robust if only because the entirety of genetic data that is considered. Based on our results, the species *V. astriarenae* formed a robust clade (bootstrap 0.99) with both *V. aestivus* and *V. mexicanus* as the closest relatives, and further formed a cluster proximal to *V. marisflavi* (bootstrap 0.95). In addition, an orphan species *Vibrio proteolyticus* defined by Sawabe et al. (2013) was clustered with *Vibrio xiamenensis* as the closest neighbor (bootstrap 0.99), and together with *Vibrio sonorensis* formed another subcluster within the clade Marisflavi. The next closest clades are Scophthalmi-Orientalis that form a deep clade within the family *Vibrionaceae* (Figure 1B and Supplementary Figure S4).

Other methological discrepancies have been described including a finding that the strain C7 was closely related to *V. agarivorans* based on analysis of MLST data, but much less so based on DNA-DNA hybridization data (Al-Saari et al., 2015). According to the latest GTDB taxonomy (r89), *V. agarivorans* was reclassified as *Vibrio sagamiensis* and has been moved to the Harveyi clade (Figure 1B), indicating the genomic divergence between these two species was much greater than expected despite sharing agarolytic activity. Interestingly, phenotypic data from our group and others has shown that all the members of the Marisflavi clade can catabolize D-galactose as a sole carbon source (Wang et al., 2011; Gao et al., 2012; Lucena et al., 2012; González-Castillo et al., 2015, 2016). It is likely that a combination of phenotypic and genotypic data can be used to provide new insights in resolving the origin and evolution of the Marisflavi clade.

We encountered some challenges with the comparisons between HN897 and C7 that were attributable to an unexpectedly highly prevalence of fragmentation or frameshifts within the coding regions of C7 assembly (see the shattered gene arrows of C7 in Figure 3C). The genome of C7 was originally assembled solely by short-read sequencing approach (Ion PGM, 400-bp reads) with low coverage (28x) (Al-Saari et al., 2015), which is expected to contribute to incomplete or fragmented genomes (Batty et al., 2018). By combining short and long-read technologies (Illumina and PacBio, respectively), we were able to assign several unassembled contigs from the original C7 based on the higher fidelity HN897 sequences (marked with asterisks in Figure 1D). For example, the first two longest protein-encoding genes in HN897 that contained dozens of tandem repeats were either not included (as for Vas2_1198) or not properly assembled (as for Vas2_433) in the C7 assembly (marked with shaded boxes in Figure 1D). Together these data demonstrated how newer sequencing technology and assembly tools can help retrieve additional insights from earlier sequencing efforts.

Many marine bacteria can be classified into two trophic modes of life namely copiotroph and oligotroph, that have evolved to thrive in distinct ocean habitats with high or low nutrient availability, respectively (Lauro et al., 2009). As one of the well-known clades of marine copiotrophs, the γ-proteobacterial order *Vibrionales* has notable potential to rapidly respond to different environmental clues (e.g., nutrient influx or depletion), which are achieved through a diverse array of metabolic pathways and fine-tuned signaling cascades (Lauro et al., 2009; Zhang X. H. et al., 2018). As mentioned earlier, the HN897 genome encodes two prominent genes Vas2_1198 and Vas2_433, which are enriched with repetitive cadherin domains (Figure 1D). Proteins containing such domains may contribute to cell–cell contact of bacteria in the marine environment (Fraiberg et al., 2010), and confer binding to different insoluble carbohydrate substrates as carbohydrate-binding modules (Fraiberg et al., 2011). The HN897 genome encoded 156 CAZymes of which eight genes likely encoded β-agarases that belonged to GH subfamilies GH16 and GH50. Synteny analysis showed that GH16s and GH50s represented two groups of tandemly arrayed genes that were likely paralogous in origin. The only exception to this was Vas1_*1339* that was located on a separate chromosome from the other GH16s.

Interspecific comparisons amongst 46 genome assemblies demonstrated the two β-agarase gene clusters of HN897 shared only partial synteny with a few Vibrios (*Vibrio* sp. EJY3; *V. celticus* CECT7224), and species of the family *Alteromonadaceae* (*Agarivorans albus*, *Agarivorans gilvus*, and *Aliagarivorans marinus*), all of which have been functionally demonstrated to be agarolytic or a dominant member of epiphytic bacterial communities associated with marine macroalgae (López-Pérez and Rodriguez-Valera, 2014; Albakosh et al., 2016; Yu et al., 2020). β-agarase genes were frequently associated with insertion sequences, integrases and transposable elements (Figure 3C) consistent with an important role for horizontal gene transfer.

We demonstrated that one of the eight β*-*agarase genes from HN897 (*Vas1_1339*) was required for agarolytic degradation (Figure 4A). Vas1_1339 belonged to the GH16 family that shared a common β-jelly roll fold and a catalytic motif (‘ExDxxE’ as shown in Figure 4C or ‘ExDxE’) within the active-site β-strand (Naretto et al., 2019; Viborg et al., 2019). Interestingly, two other *Vibrio* β-agarases, namely AgaA from *Vibrio* sp. PO-303 (Dong et al., 2007), and AgaV from *Vibrio* sp. V134 (Zhang and Sun, 2007) were assigned with Vas1_1339 to the subfamily GH16_16. AgaV is an endo-acting β-agarase that degrades agarose into neoagarotetraose (NeoDP4) and NeoDP6 (Zhang and Sun, 2007).

None of the remaining seven β*-*agarase genes from HN897 appeared to contribute to agarolysis activity of HN897. Three of them [Vas2_922(WP_164650518.1), Vas2_927(WP_164650523.1), Vas2_928] were recently classified into subfamily GH16_17 according to the latest CAZy database (update: 2020-05-01). GH16_17 is a small group containing carbohydrate-active κ-carrageenases (EC 3.2.1.83) from both Proteobacteria and Bacteroidetes (Viborg et al., 2019), yet only one Vibrio-originated CgkA (QGN18698.1; partial) from *Vibrio* sp. SY01 is recorded in addition to the three from this study. Nevertheless, a recent study identified a κ/ι-carrageenan metabolism pathway in several marine *Pseudoalteromonas* species, with a κ/ι-carrageenan-specific polysaccharide utilization locus (CarPUL) responsible for this phenotype (Hettle et al., 2019). Within the CarPUL of *Pseudoalteromonas fuliginea*, three GH16_17-coding genes namely GH16A (EU509_08815), GH16B (EU509_08870), and GH16C (EU509_08960) were separated with several intervening genes including sulfatase and major facilitator superfamily (MFS) transporter genes. Interestingly, the GH16_17s from HN897 shared 40–57% sequence identity with those of *P. fuliginea.* Given that both HN897 and C7 have orthologous genes associated with carrageenan metabolism (see Supplementary Table S5; Hettle et al., 2019), it was tempting to speculate that *V. astriarenae* has the ability to metabolize galactan carrageenan.

The GH50 family of proteins is smaller than the GH16 family, and is considered monospecific to agarose (Kim et al., 2010). To date, eight of eleven *Vibrio*-originated proteins with known β-agarase activity are classified as GH50s (Chi et al., 2012; Yu et al., 2020). For example, The *Vibrio* strain EJY3 has three tandemly arranged GH50s (VejGH50A, VejGH50B, and VejGH50C) that encode extracellular endo-type β-agarases responsible for degrading agarose into neoagarotetraose (NeoDP4) and NeoDP2. Strian EJY3 also has one exo-type β-agarase (VejGH50D) that acts intracellularly to depolymerize NeoDP4 into monomeric sugars (Yu et al., 2020). While most agarose saccharification (agarolysis) appears to involve initial extracellular depolymerization by endo-type β-agarase before transport into the cell (Pluvinage et al., 2013; Yu et al., 2020), such ‘pre-depolymerization’ may not be necessary for EJY3, considering the absence of both GH16 and GH86 in its genome (Roh et al., 2012). In contrast, AgaV from *V. astriarenae* strain V134 can decompose agarose into NeoDP4 and NeoDP6, besides, additional larger intermediate products including NeoDP8 and NeoDP10 can be also observed (Zhang and Sun, 2007). Consequently, we proposed that β-agarase-mediated agarose degradation in *V. astriarenae* was composed by two steps. This begined with degradation of agarose into medium-sized DPs (e.g., NeoDP4 and NeoDP6) based on activity of GH16_16 (Vas1_1339) before both secreted endo-type and intracellular exo-type GH50 β-agarases alongside with other CAZymes, as collectively characterized in the strain EJY3, hydrolyze the DPs to yield monomeric sugars as a carbon source. Follow-up studies are needed to functionally characterize these agar-specific polysaccharide utilization loci and clarify the in-depth biochemical pathways of agarose degradation in *V. astriarenae* HN897.

In summary, we presented here the high-quality genomic sequence of an agarolytic *V. astriarenae* strain HN897, which had an abundance of putative glycoside hydrolases (GH). Eight putative β-agarase encoding genes belonging to the GH16 and GH50 families were further identified based on sequence clustering and comparisons of protein domain architecture. Importantly, GH16_16 β-agarase-coding gene *Vas1_1339* was indispensable for the agarolytic phenotype of HN897, as evidenced by gene deletion and complementation experiments. These results not only provided the basis for understanding the biochemical mechanism of bacterial agarolysis, but also for gaining more detailed insights into the evolution and physiological capability of the genome of *V. astriarenae* HN897.

## Data Availability Statement

The datasets generated for this study can be found in the NCBI GenBank under accession numbers CP047475 (Vas1) and CP047476 (Vas2).

## Author Contributions

YL, CW, XZ, and ML carried out the experiments. XJ and ZZ designed the experiments. XJ and YL analyzed the data. ZZ, XJ, and YL wrote the manuscript. DC contributed to phylogenetic analysis and manuscript preparation. All the authors have read and agreed to the published version of the manuscript.

## Conflict of Interest

The authors declare that the research was conducted in the absence of any commercial or financial relationships that could be construed as a potential conflict of interest.

## Footnotes

1 https://www.genome.jp/tools/kaas/

2 http://www.kegg.jp

## Supplementary Material

FIGURE S1 Two CG maps generated with the GView Server showed the full view of the chromosomes Vas1 **(A)** and Vas2 **(B)** of HN897, respectively. The contents of the feature rings (starting with the outermost ring) are as follows: Rings 1,2: positive stranded and Rings 3,4: negative stranded CDSs that were color-coded with COGs and RNA species; Ring 5 shows GC content and Ring 6 shows GC skew.

FIGURE S2 Functional annotations of HN897 genome by COGs. Bar plot showed the numbers of genes that had been classified with each indicated COG group.

FIGURE S3 Species counts and sequence alignment statistics of top BLAST hits for the genome sequence of *V. astriarenae* HN897. Pie charts showed the composition of hit species of homologous protein sequences aligned by BLASTp against the non-redundant (nr) protein database for Vas1 **(A)** and Vas2 **(B)**. Bar plots showed the average percentage of identity and coverage of sequence alignment (alignment length divided by query length) of Vas1 **(C)** and Vas2 **(D)** against each indicated species.

FIGURE S4 Full view of the maximum-likelihood species tree shown in Figure 1B. The bootstrap support (0∼1) were labeled in each branch node.

FIGURE S5 ML phylogenetic tree of 16S rRNA gene were estimated by FastTree2 using GTR + gamma model, based on the MAFFT-aligned sequences from Al-Saari et al. (2015) and other Vibrios which have high sequence similarity (>98%) to HN897. Strains that had been referred in the texts were highlighted with red. *V. astriarenae* clade was shaded in gray. Sequence from *E. coli* K-12 substrain DH10B was use as outgroup.

FIGURE S6 Multiple sequence alignment of full-length Vas1_1339 with orthologous bacterial β-agarases (see Supplementary Table S7 for full sequence list). The secondary structure information was based on resolved 3D structure of ZgAgaB (Protein Data Bank: 1o4z) from *Zobellia galactanivorans*. η-(3_10_) and α-Helices were shown as helices; β-Strands were shown as arrows; and β-Turns were labeled with TT. The figure was drawn by ESPript 3.0 (Robert and Gouet, 2014).

TABLE S1 Bacterial strains and plasmids used in this study.

TABLE S2 Sequences for oligo primer pairs used in each indicated experiment.

TABLE S3 Phylogenomics and BLAST sequence alignments. tab#1: Data related to Supplementary Figures S3A–D. Annotation output from BLASTP-NR-based sequence similarity comparisons for both Vas1 and Vas2. tab#2: Data related to Figures 1A,B. The full standard metrics for HN897 assembly according to the Genomic Standards Consortium (GSC). Taxonomic classification and genome completeness assessment for Vibrio astriarenae HN897 that were performed by GTDB-tk ‘classify_wf’ and CheckM, respectively. tab#3: Data related to Figure 1B and Supplementary Figure S4. Metadata of 140 representative *Vibrio* genomes from GTDB release 89. tab#4: Data related to Figures 1C,D. tBLASTx hits between HN897 and C7, with threshold of Evalue 1e-10, alignment length 100, percent identity 90.

TABLE S4 Functional annotations of HN897 genome. tab#1: Related to Figure 2A. Output from KAAS-based KEGG Orthology assignment using BLASTp with BBH (bi-directional best hit) method and against representative prokaryotes genes set. tab#2: Related to Figure 2A. KEGG pathway maps sorted by count numbers of associated KO terms resulted from KEGG mapper searching. tab#3: Related to Figure 2B. Protein domain modules annotated by PfamScan v1.6 searching against Pfam32.0 database. tab#4: Related to Figure 2B. Protein domain list sorted by count numbers of domain terms (shortname). tab#5: Related to Figure 2B. Gene Ontology annotations for Top 30 counted domain terms. tab#6: Related to Figure 2C. Gene counts for each indicated class of carbohydrate-active enzymes, CAZymes, for Vas1 and Vas2. tab#7: Related to Supplementary Figure S2. Count numbers of associated genes annotated with indicated categories of Clusters of Orthologous Genes (COGs). tab#8: Related to Supplementary Figure S3A. Proteins sequences for a total of 156 identified CAZymes in HN897 genome.

TABLE S5 Identification of the β-agarase gene repertoire in HN897. tab#1: Related to Figure 3A. Annotations for a total of 72 identified GH subfamilies in HN897 (Eight putative beta-agarase were highlighted). tab#2: Related to Figures 1C,D, 3A,B. InterProScan outputs for the protein domains of 72 Glycoside Hydrolases, together with the first two longest CDSs that contain the tandem repeat sequences discussed in manuscript. tab#3: Related to Figure 3A. Generic Feature Format Version 3 (GFF3) file of the catalytic protein domains for a total of 72 GHs annotated by CAZy database. tab#4: Related to Figure 3C. A total of 45 subject genome assemblies used for sequence similarity searching of β-agarase gene cluster. tab#5: Related to Figure 3C Upper panel: The output of MultiGeneBLAST v1.1.14 concerning the comparions of β-agarases containing regions on Vas1 (Four GH50s and a single GH16 Vas1_1339) among the investigated 46 genomes. tab#6: Related to Figure 3C Bottom panel: The output of MultiGeneBLAST v1.1.14 concerning the comparions of β-agarases containing regions on Vas2 (Three GH16s) among the investigated 46 genomes.

TABLE S6 Related to Supplementary Figure S5. Sequences used for constructing phylogenetic tree of 16S rRNA gene, that were retrieved according to Al-Saari et al. (2015) and other Vibrios which have high sequence similarity (>98%) to HN897.

TABLE S7 Related to Figure 4C and Supplementary Figure S6. Top hit similar sequences (screened by coverage and identity) to Vas1_1339 aligned by BLASTp against non-redundant (nr) GenBank database.
