# Supplementary figures and images for: Genome-Wide Identification and Functional Characterization of β-Agarases in Vibrio astriarenae Strain HN897
Source: Front Microbiol. 2020 Jun 24;11:1404. doi: 10.3389/fmicb.2020.01404 (PMC7326809; doi:10.3389/fmicb.2020.01404)

A

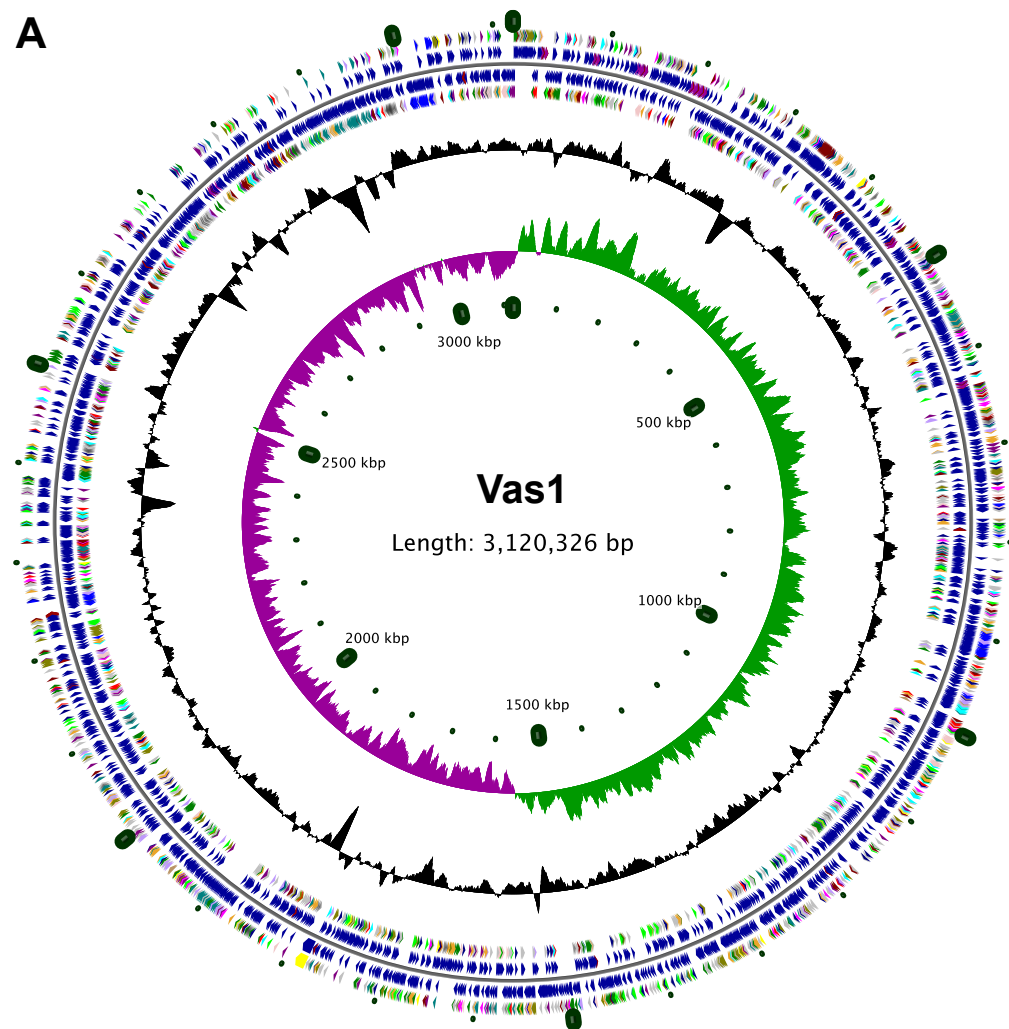

B

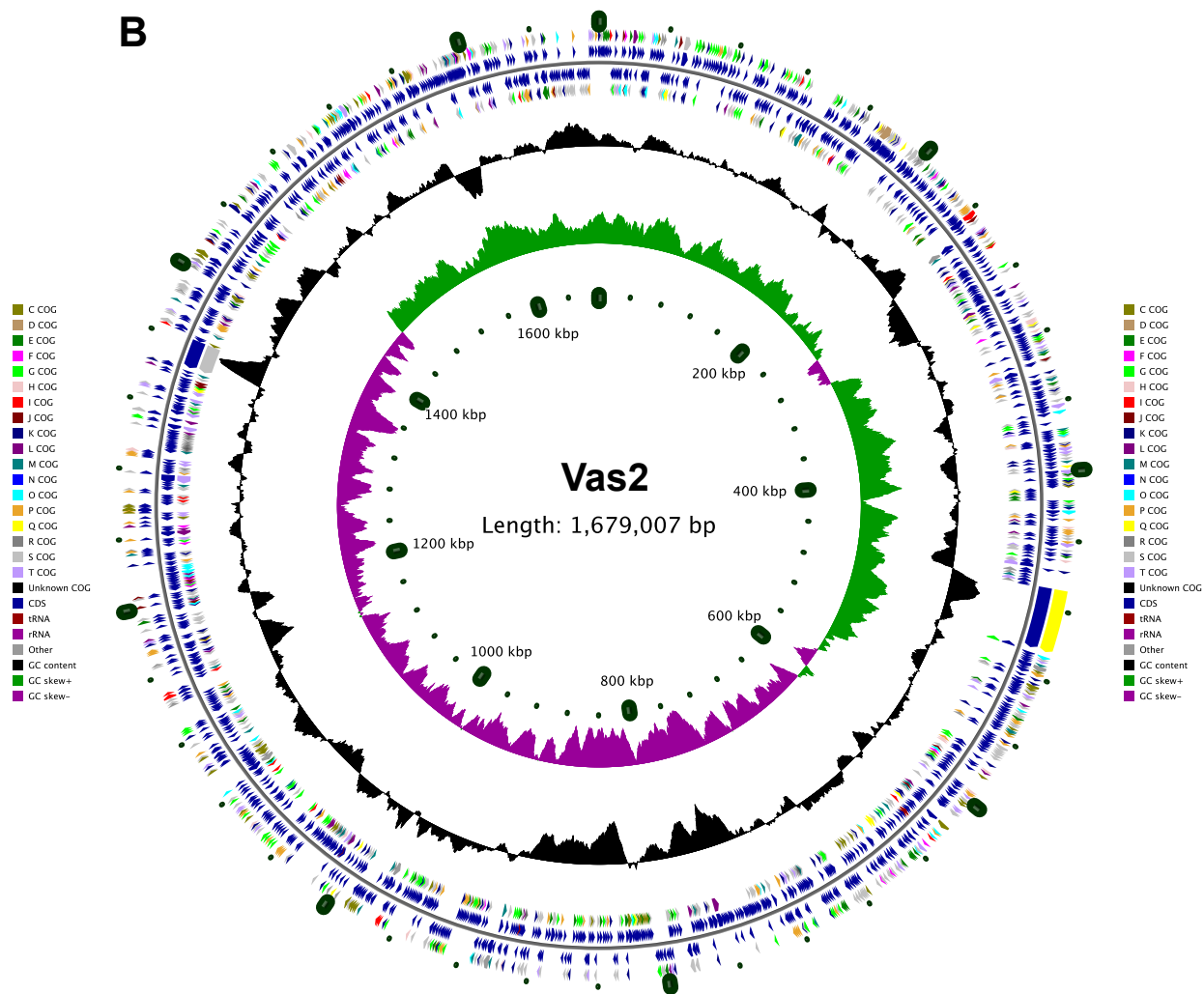

Supplement: FIGURE S1 — Two CG maps generated with the GView Server showed the full view of the chromosomes Vas1 (A) and Vas2 (B) of HN897, respectively. The contents of the feature rings (starting with the outermost ring) are as follows: Rings 1,2: positive stranded and Rings 3,4: negative stranded CDSs that were color-coded with COGs and RNA species; Ring 5 shows GC content and Ring 6 shows GC skew. [file Image_1.pdf]

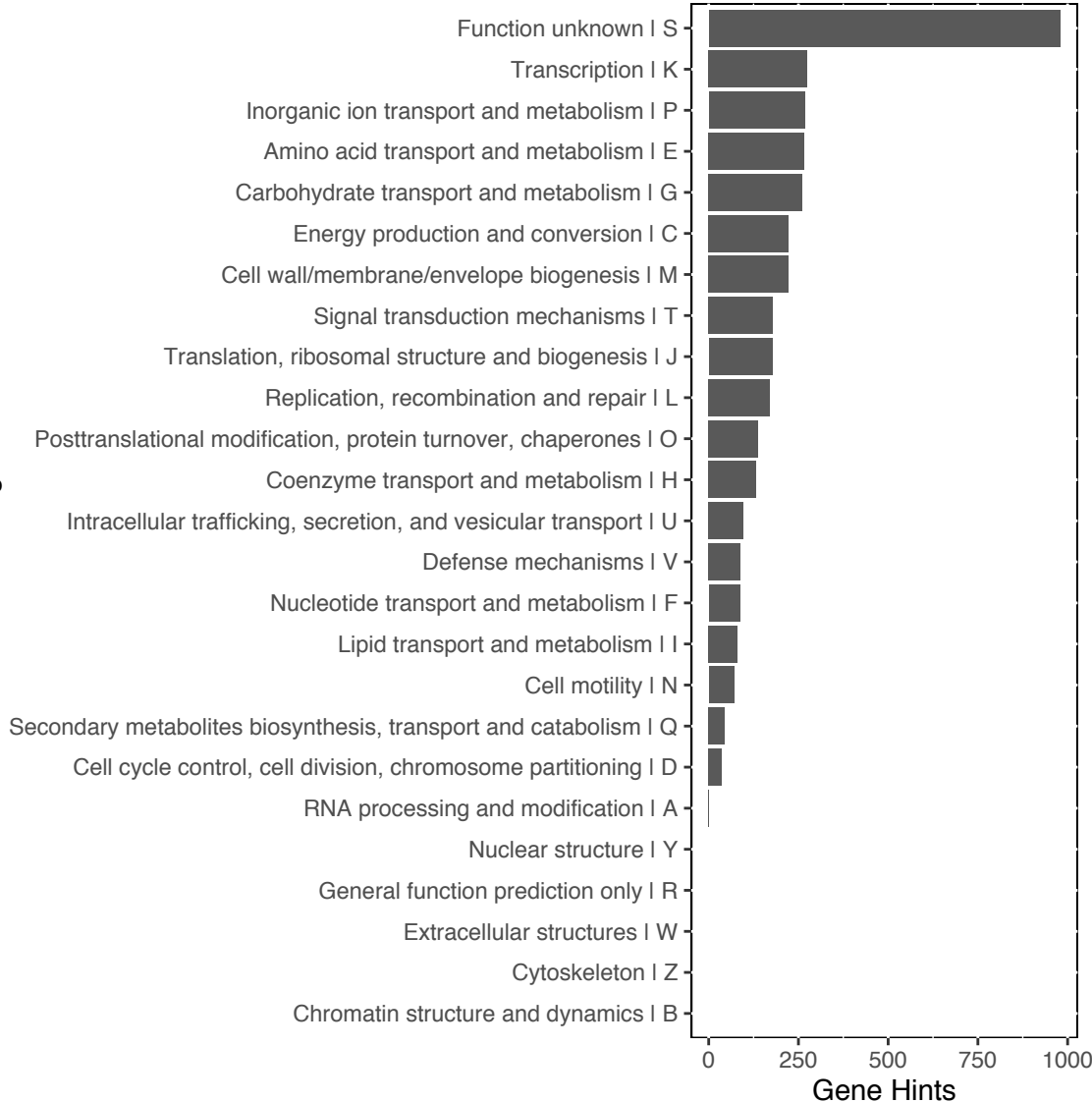

Supplement: FIGURE S2 — Functional annotations of HN897 genome by COGs. Bar plot showed the numbers of genes that had been classified with each indicated COG group. [file Image_2.pdf]

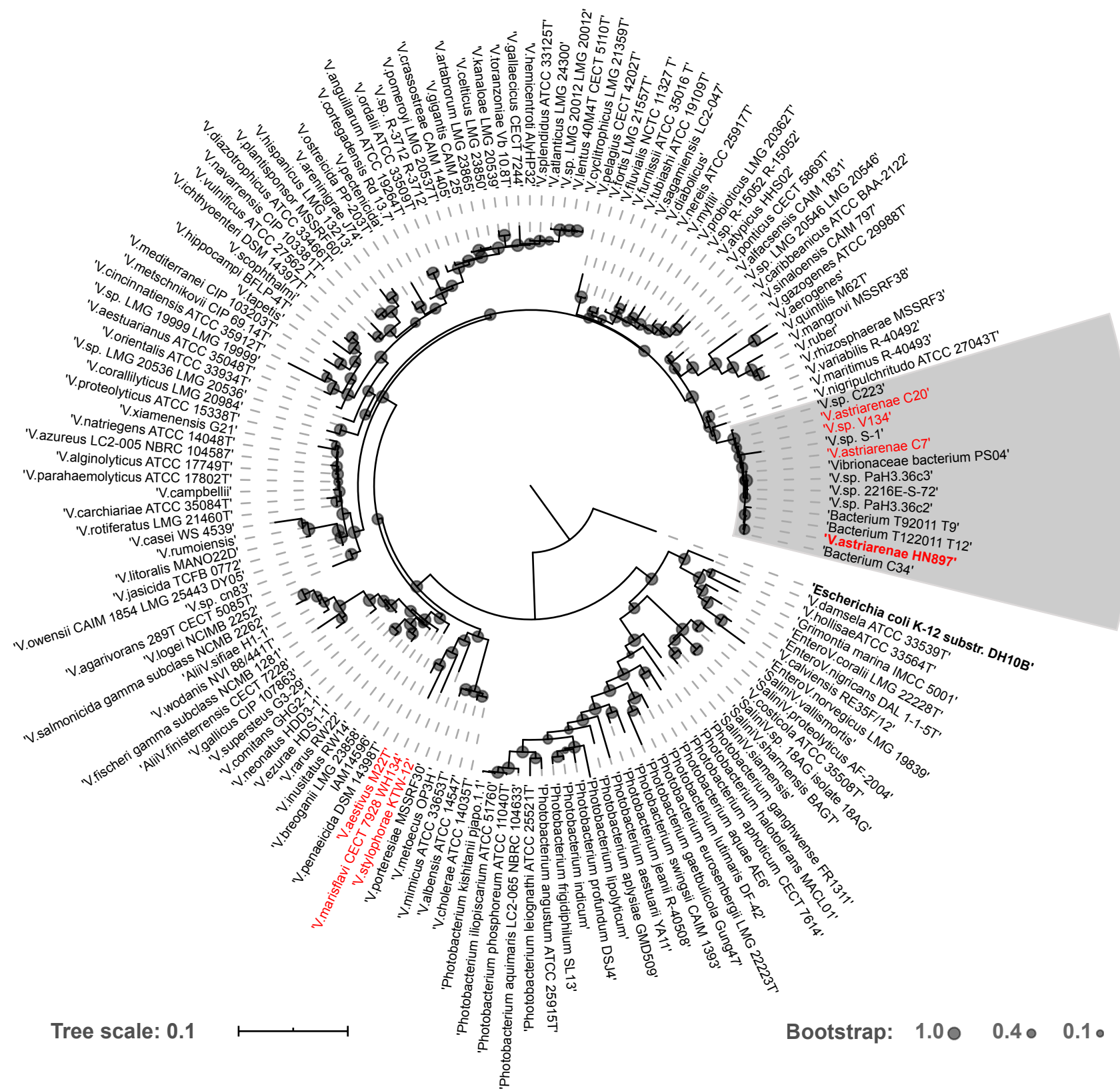

Supplement: FIGURE S5 — ML phylogenetic tree of 16S rRNA gene were estimated by FastTree2 using GTR + gamma model, based on the MAFFT-aligned sequences from Al-Saari et al. (2015) and other Vibrios which have high sequence similarity (>98%) to HN897. Strains that had been referred in the texts were highlighted with red. V. astriarenae clade was shaded in gray. Sequence from E. coli K-12 substrain DH10B was use as outgroup. [file Image_5.pdf]
