# Supplementary material for: Genome-Wide Identification and Functional Characterization of β-Agarases in Vibrio astriarenae Strain HN897
Source: Front Microbiol. 2020 Jun 24;11:1404. doi: 10.3389/fmicb.2020.01404 (PMC7326809; doi:10.3389/fmicb.2020.01404)
Supplement: FIGURE S3 — Species counts and sequence alignment statistics of top BLAST hits for the genome sequence of V. astriarenae HN897. Pie charts showed the composition of hit species of homologous protein sequences aligned by BLASTp against the non-redundant (nr) protein database for Vas1 (A) and Vas2 (B). Bar plots showed the average percentage of identity and coverage of sequence alignment (alignment length divided by query length) of Vas1 (C) and Vas2 (D) against each indicated species. [file Image_3.pdf]

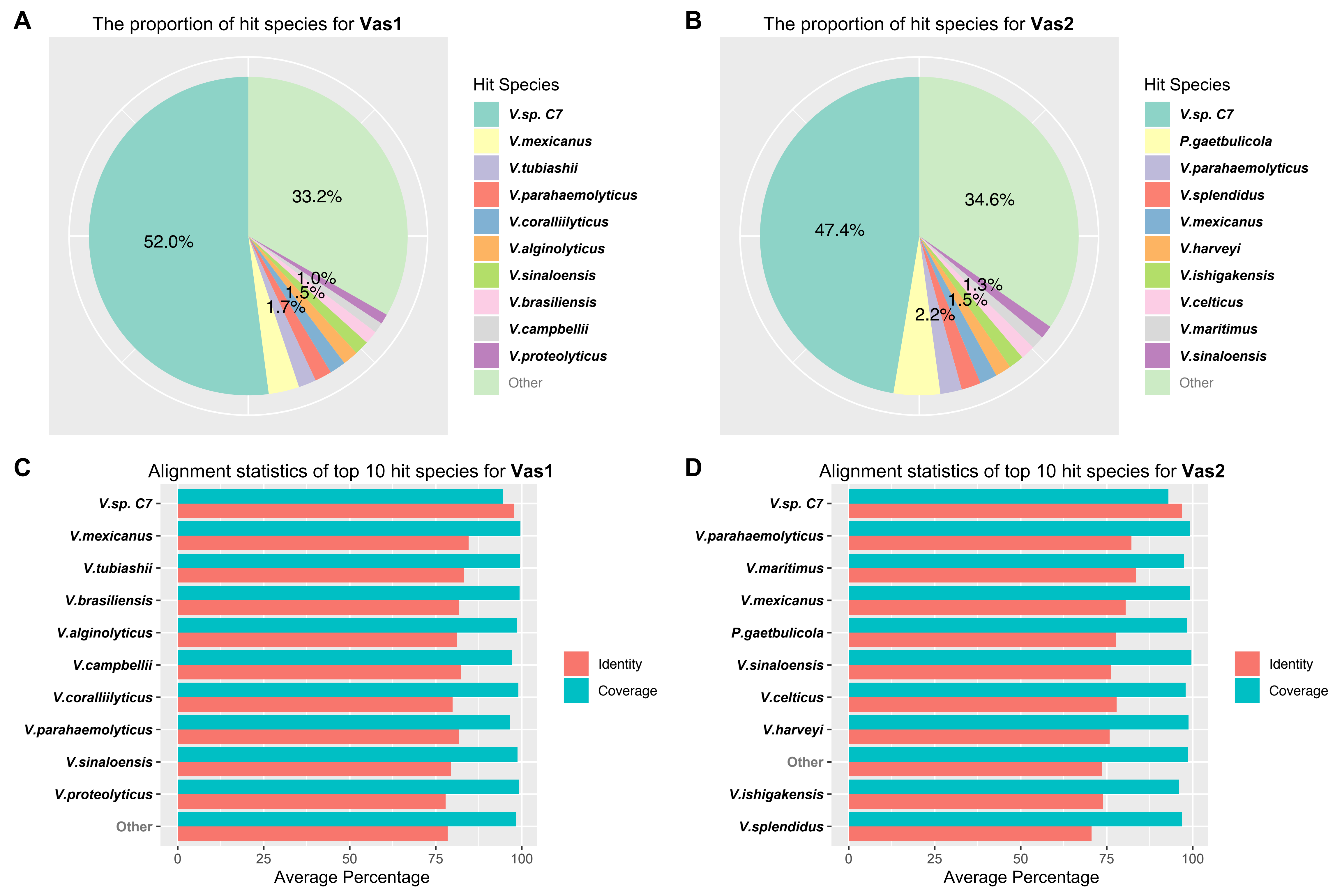

A
The proportion of hit species for Vas1
52.0%
33.2%
1.0%
1.5%
1.7%
Hit Species
V.sp. C7
V.mexicanus
V.tubiashii
V.parahaemolyticus
V.coralliilyticus
V.alginolyticus
V.sinaloensis
V.brasiliensis
V.campbellii
V.proteolyticus
Other
B
The proportion of hit species for Vas2
47.4%
34.6%
1.3%
1.5%
2.2%
Hit Species
V.sp. C7
P.gaetbulicola
V.parahaemolyticus
V.splendidus
V.mexicanus
V.harveyi
V.ishigakensis
V.celticus
V.maritimus
V.sinaloensis
Other
C
Alignment statistics of top 10 hit species for Vas1
V.sp. C7
V.mexicanus
V.tubiashii
V.brasiliensis
V.alginolyticus
V.campbellii
V.coralliilyticus
V.parahaemolyticus
V.sinaloensis
V.proteolyticus
Other
0
25
50
75
100
Average Percentage
Identity
Coverage
D
Alignment statistics of top 10 hit species for Vas2
V.sp. C7
V.parahaemolyticus
V.maritimus
V.mexicanus
P.gaetbulicola
V.sinaloensis
V.celticus
V.harveyi
Other
V.ishigakensis
V.splendidus
0
25
50
75
100
Average Percentage
Identity
Coverage
