# Supplementary material for: Genome-Wide Identification and Functional Characterization of β-Agarases in Vibrio astriarenae Strain HN897
Source: Front Microbiol. 2020 Jun 24;11:1404. doi: 10.3389/fmicb.2020.01404 (PMC7326809; doi:10.3389/fmicb.2020.01404)
Supplement: FIGURE S4 — Full view of the maximum-likelihood species tree shown in Figure 1B. The bootstrap support (0∼1) were labeled in each branch node. [file Image_4.pdf]

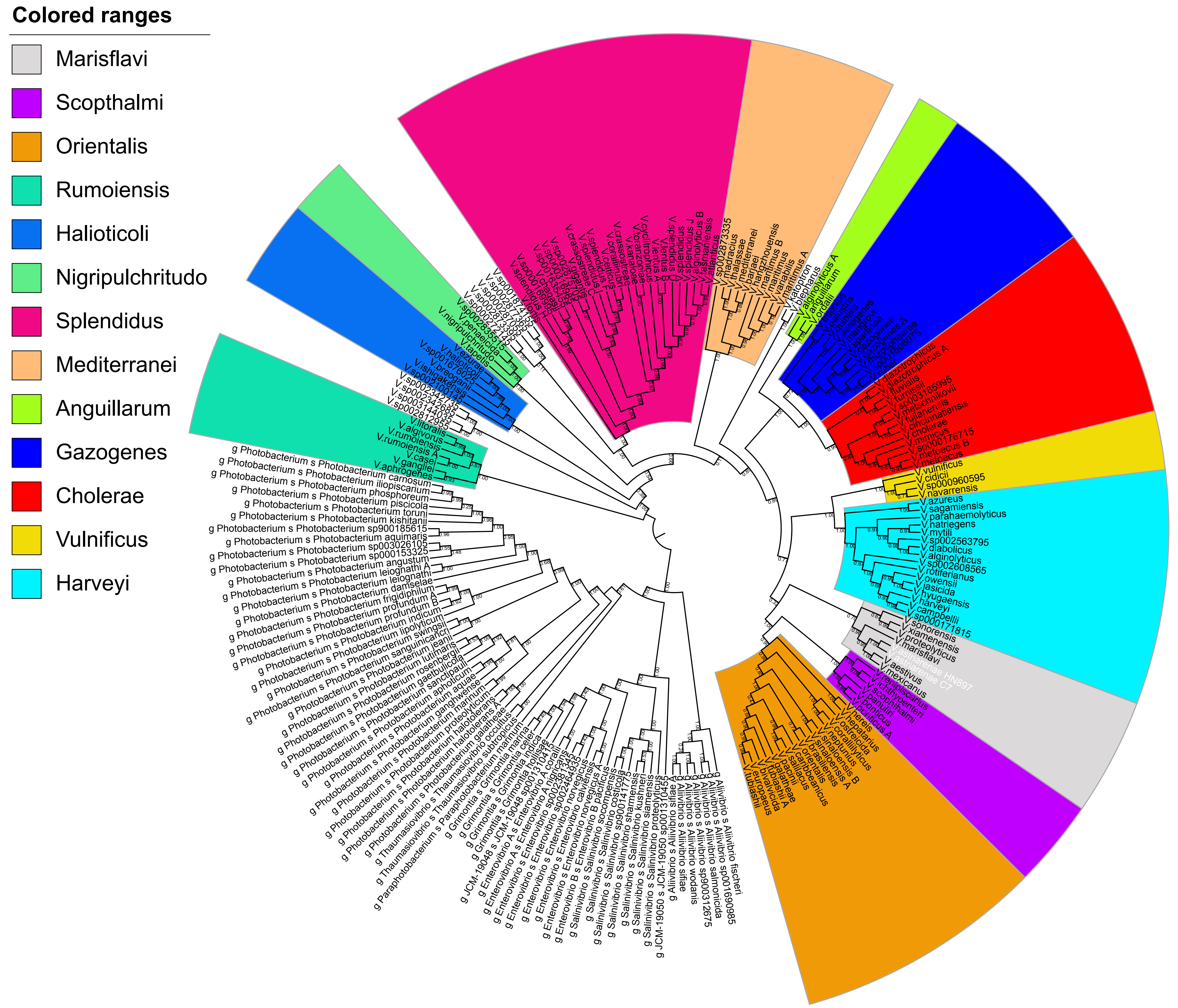
Colored ranges
Marisflavi
Scopthalmi
Orientalis
Rumoiensis
Halioticoli
Nigripulchritudo
Splendidus
Mediterranei
Anguillarum
Gazogenes
Cholerae
Vulnificus
Harveyi
