# Supplementary material for: Genome-Wide Identification and Functional Characterization of β-Agarases in Vibrio astriarenae Strain HN897
Source: Front Microbiol. 2020 Jun 24;11:1404. doi: 10.3389/fmicb.2020.01404 (PMC7326809; doi:10.3389/fmicb.2020.01404)
Supplement: FIGURE S6 — Multiple sequence alignment of full-length Vas1_1339 with orthologous bacterial β-agarases (see Supplementary Table S7 for full sequence list). The secondary structure information was based on resolved 3D structure of ZgAgaB (Protein Data Bank: 1o4z) from Zobellia galactanivorans. η-(310) and α-Helices were shown as helices; β-Strands were shown as arrows; and β-Turns were labeled with TT. The figure was drawn by ESPript 3.0 (Robert and Gouet, 2014). [file Image_6.pdf]

```
1o4z
                   1         10        20        30        40        50
1o4z               ...MYLIYLRLVFCCALLLGCGDNSKFDSATDLPVEQEQEQETEQEGEPEESSEQDLVEE
Agaribacterium     .......MNKHSLACLTALSVL...........................SFSPTALVNA
Shewanella         MKGKYMKHHLNLVCATALLSTS..........................VLSSS..IYA
Shewanella         .....MKPSMNVIYATTLMATS..........................ALTTV..CIA
Pseudoalteromonas  ...........MNKTTLFIGCL..........................LTTTN..LFA
Colwellia          ............MKKTSVLLTG..........................IIFTSFIGHT
Catenovulum        ............MKKLALLAIS..........................SVFST.SGIA
Gayadomonas        ............MKHLSALITG..........................LLLTT.TAHS
Alteromonas        ............MKNLSILLAG..........................IITST.VAHC
Aliagarivorans     .........MFNVQRVLISAVS..........................LSVCS.AVQA
Agarivorans        .......MLPRHYKNLLLTQG...........................LSLSFASLAA
Agarivorans        ........MKGFTKHPLLACG...........................LGLSL.STYA
Vibrio             .............MKLPILSLL..........................TIAIN.PAFA
Vas1_1339          ........MKSIIKTLTLLPIF..........................LTSTT..LMA
Vibrio             ........MKSTIKSLPLLPFF..........................LMSTS..LMA

                     η1              β1                       α1    β2          β3
1o4z                             TT-----> T.T          TT                    TT--
                   60        70        80        90        100       110
1o4z               VDWKDIPVPADAGPNMKWEFQE.ISDNFEYEAPADNKGSEFLEKWDDFYHNAWAGPGLTE
Agaribacterium     YDWDNVAIPAYAGDGKRWQLQPQYSDDFNYSG....KNATFHSKWKDGYFNNWTGPGLTY
Shewanella         ADWDNIPLPAVAGNGLVWQLNDQYSDSFNYNG....KTSTFTSKWNDTYFNAWEGPGLTR
Shewanella         ADWDNIPLPADPGNGLVWQLNEQYSDSFNYQG....KNATFNSKWNDTYFNNWEGPGLTR
Pseudoalteromonas  NDWDSIPLPVTPGDGKVWQLQETYSDSFNYTG....KPAAFTSKWNDTYFNSWTGPGLTY
Colwellia          ADWDNIPVPAAAGAGKSWELQSSYSDSFNYTG....KPNTFTSKWNDTYFNGWTGPGLTY
Catenovulum        ADWDGIPVPASAGAGKTWQLQDSYSDSFNYTG....KPSSFTNKWKDTYFNGWTGPGLTH
Gayadomonas        EDWDNVPIPASPGAGKVWQLQTQYSDSFNYTG....KPNQFTSKWNDSYFNNWSGPGLTH
Alteromonas        EDWDNIPLPTSPGGGKVWQLQTQYSDSFNYIG....KPSDFTDKWNDSYFNNWTGPGLTY
Aliagarivorans     NDWDDIPIPADPGSGYVWELQEAYSDSFNYTG....KPSAFSSKWNDTYFHGWTGPGLTY
Agarivorans        NDWDTIPIPANPGTGYVWELQEAYSDSFNYSG....KTNEFTSKWNDSYFKSWTGPGLTH
Agarivorans        ADWDNIPIPAELDPGQSWELQESYSDSFNYSG....KPSSFTSKWKDAYFHNWTGPGLTY
Vibrio             NDWDSIPIPAELDDGQQWELQEAYSDSFNYTG....KNTTFTSKWNDTYFHGWTGPGLTY
Vas1_1339          EDWREIPIPAPLEDGQSWELQEAYSDSFNYTG....KGDSFRSKWNDTYFHGWTGPGLTY
Vibrio             NDWQDIPIPAPLDDGQSWELQEAYSDSFNYTG....KGDEFSSKWNDTYFHGWTGPGLTY

                     η2  β4       β5    β6       β7   β8         β9             β10
1o4z               >        ---->TT---->  -->  TT   -->  --->      --------->    ------
                     120       130       140       150       160       170
1o4z               WKRDRSYVADGELKMWATRKPGSDKINMGCITSKTRVVYPVYIEARAKVMNSTLASDVWL
Agaribacterium     WKKDHSNVAGGALVLRASRH.GSDRVGTGVITSKTKVTYPIFMEARIKVSNLELSSNFWL
Shewanella         WSSGESWVSDGNLIISASRK.APDLVNAGVITSKTKVKYPIFLEANIKVSNLELSSNFWL
Shewanella         WSQNESWVSDGNLIISASRK.APNLVNAGVITSKTKVKYPIFLEANIKVSNLELSSNFWL
Pseudoalteromonas  WQQDESWVSDGNLIISASRRAGTDKVNAGVITSKTKVSFPIFLEANIKVSNLELSSNFWL
Colwellia          WQKNSSWVSDGNLIISASRKAGTNKVNAGVITSKTKVKFPIFLEANIKVSNLELSSNFWL
Catenovulum        WSSSESWVSDGNLIISASRRQGTNKVNAGVITSKTKVKYPIFMEARIKVSNLELSSNFWL
Gayadomonas        WSSNESWVADGNLIISASRRQGTNKVNAGVITSKTKIKYPVYLEARIKVSNLELSSNFWL
Alteromonas        WSSNESWVANGNLIISASRRQGTNQVNAGVITSKTKVKFPIYLEARIKVSNLELSSNFWL
Aliagarivorans     WSSDESWVSDGNLIISASRRAGTNQVNAGVVTSKTKVKYPIFLEASIKVSNLELSSNFWL
Agarivorans        WDSSESWVADGNLIVSASRRQGTDKVNAGVITSKTKVKYPIFLEASIKVSNLELSSNFWL
Agarivorans        WSSDESWVGDGNLIISASRRQGTNQVNAGVVTSKTKVKYPIFLEANIKVSNLELSSNFWL
Vibrio             WQSNESWVSDGNLIISASRRQGTEQVNAGVVTSKTKVKYPIFMEARIKVSNLELSSNFWL
Vas1_1339          WQSDESWVDDGNLIISASRRQGTEMVNAGVVTSKTKVKYPIFLEANIKVSGLELSSNFWL
Vibrio             WQRDESWVDDGNLIISASRRQGTEMVNAGVVTSKTKVKYPIFLEANIKVSGLELSSNFWL

                            β11        β12   β13  α2        β14              β15
1o4z               -->TT    --------->  -->  -->          --------->           --->
                     180       190       200       210       220
1o4z               LSADDTQEIDILEAYGADYSESAGKDHSYFSKKVHISHHVFIRD....PFQDY..QPKD
Agaribacterium     LSDNDQREIDILEVYG.......GARDSWFAKNMSTNFHVFFRNSDNS.IKSDYNDQTHN
Shewanella         LSENDEREIDILEVYG.......GARETWFAQNMSTNFHVFLRNGDNS.IKSDFNDQTHN
Shewanella         LSENDEREIDILEVYG.......GSRETWFTQNMSTNFHVFLRNSDNS.IRSDFNDQTHN
Pseudoalteromonas  LSDNDEREIDVLEVYG.......GARDDWFAKNMSTNFHVFIRDQQSNQIISDYNDQTHN
Colwellia          LSENDEREIDVLEVYG.......GARETWFAKNMSTNFHVFVRDEQSNEIISDFNDQTHN
Catenovulum        LSENDEREIDILEVYG.......GASDTWFAKNMSTNFHVFFRNNQTNAIISDFNDQTHN
Gayadomonas        LSENDEREIDILEVYG.......GASDTWFAKNMSTNFHVFFRDQQTNAIISDYNDQTHN
Alteromonas        LSQNDEREIDILEVYG.......GAADTWFAKNMSTNFHVFLRDEQTNQIISDFNDQTHN
Aliagarivorans     LSENDQREIDILEVYG.......GAEDEWFAKNMSTNFHVFFRDGGNN.IISDFNDQTHN
Agarivorans        LSENDEREIDILEVYG.......GAADTWYAKNMSTNFHVFIRDAASNQIISDFNDQTHN
Agarivorans        LSENDQREIDVLEVYG.......GARQDWFAKNMSTNFHVFFRNNDNS.ISSDFNDQTHN
Vibrio             LSENDQREIDILEVYG.......GAKDTWFAKNMSTNFHVFLRNSDNT.IKSDFNDQTHN
Vas1_1339          LSENDQREIDVLEVYG.......GAEDEWFAKNMSTNFHVFFRNEDNS.IRSDFNDQTHN
Vibrio             LSENDEREIDVLEVYG.......GAEDEWFARNMSTNFHVFFRNDDNT.IRSDFNDQTHN

                     η3 β16      η4     β17          β18         β19      α3        TT      TT
1o4z                     -->            --------->TT------->TT--------->
                   230       240       250       260       270       280
1o4z               AGSWFEDGTVWNKEFHRFGVYWRDPWHLEYYIDGVLVRTVSGKDII.DPKHFTNTTDPGN
Agaribacterium     QSN....GYWRDGYHRFGAYWKSPSDVTFYIDGKKTPDGSWAQAEMFDKDYT.....RT
Shewanella         TPVW...GNTWREGFHRFGAYWKSPTDVTFYIDGQETPNGSWDEVVMKDKDYT.....GA
Shewanella         TPTW...GNYWREGFHRFGAYWKSPTDVTFYIDGQETPQGSWAEVVMKDKDYT.....GA
Pseudoalteromonas  TPSW...GTYWREGFHRFGVYWKSPTEVTFYIDGQQTPDGSWAQVVMKDKDYT.....GA
Colwellia          TPTW...GTHWRDDFHRFGVYWKSPTEVTFYIDGQQTNEGSWAQVIMKDKDFT.....GA
Catenovulum        TPPW...GTYWRDGFHRFGAYWKSPTEVTFYINGQQTSDGSWAQAVMKDKDYT.....GA
Gayadomonas        EPAT...GTYWREQFHRFGAYWKSPTDVTFYIDGQQTPDGSWAQVVMKDKDYT.....GA
Alteromonas        IPST...GTYWRDQFHRFGAYWKSPTEVTFYIDGQQTPDGSWAQVVMKDKDYT.....GA
Aliagarivorans     TPSW...GTYWRDGFHRFAAYWKSPTDVTFYINGQQTPEGSWAQVVMKDKDYT.....GA
Agarivorans        EPSW...GTYWRDGFHRFAAYWKSPTEVTFYIDGQQTPEGSWAQVVMKDKDYT.....GA
Agarivorans        TPTW...GNYWREGFHRFGVYWKSPTEVTFYINGQKTTKGAWSQVVMKDKDYT.....GA
Vibrio             EPSW...GTYWRDGFHRFAAYWKSPTEVTFYIDGVKTPKGSWEQVLMKDKDYT.....GQ
Vas1_1339          EPQW...GTYWRDGFHRFAVYWKSPTEVTFYINGIETPKGSWEDVVMKDKDYT.....GA
Vibrio             EPQW...GTYWRDGFHRFAVYWKSPTEVTFYINGIETPKGSWEDVIMKDKDYT.....GA
```

```
                    TT                 β20         α4       TT      η5       α5     η6         β21
1o4z               290          300       310          320        330        340
1o4z               TEIDTRTGLNKEMDIIINTEDQTWRSSPASGLQSNTYTPTDNELSNIENNTFGVDWIRIY
Agaribacterium     YLDKSRYNMNQSMFMILDMEDHEWRSRAG.......NVASDADLADNSKNRMYVDWVRTY
Shewanella         IMNKSQYTMDEEMFIILDTEDHSWRSEAG.......NIATNADLADASKNKMYVDWIRVY
Shewanella         IMDKNQYTMNEEMFIILDTEDHSWRSEAG.......NVATDADLADQSKNKMYVDWIRVY
Pseudoalteromonas  TLNKNTHNMDQSAYIIIDTEDHDWRSEAG.......NIATDADLADGSKNKMYVDWVRVY
Colwellia          ILDKSQSNMDEEAFIIIDTEDHSWRSEAG.......HVATDTDLADNKKNKMYVDWIRVY
Catenovulum        ILDKSQYNMDEEMFIILDTEDHDWRSNQG.......IVASDADLANGNKNKMYVDWIRVY
Gayadomonas        ILDKSQYNMDQETYIILDTEDHEWRSNQG.......IVASDSDLANPSKNKMYVDWIRVY
Alteromonas        TLDKSQYNMDQEAFIIIDTEDHDWRSNQG.......IVASDAELADGSKNKMYVDWIRVY
Aliagarivorans     ILDKSRYNMDEEMFIILDTEDHSWRSEQG.......IVASDADLANPNKNKMYVDWIRVY
Agarivorans        VLDKSVYNLNQEAFIIIDTEDHSWRSEMG.......IIASDTDLADNSKNKMYVDWVRVY
Agarivorans        ILDKSRYNMDQEMFIILDTEDHSWRSEAG.......HIATDADLADGDKNKMYVDWIRVY
Vibrio             TLDKSQFNMDEEMFIILDTEDHSWRSEAG.......IVASDADLADSSKNKMYVDWIRVY
Vas1_1339          ILDKSTYNMDQEMFIILDTEDHSWRSEAG.......IIAKDEDLADDSKNKMYVDWIRVY
Vibrio             ILDKSTYNMDEEMFIILDTEDHSWRSEAG.......IVAKDEDLADNSKNKMYVDWIRVY

1o4z
                   350
1o4z               KPVEK.......................................................
Agaribacterium     KPVASGD.GGPSGG..TTLPPNDWTNLKMRHSAKCMDVAYGARTNGSTYHQWACNQNNVN
Shewanella         QPVSDGSSGGNTGG..N.INPNTQYQVVAKHSNKCVDVIDSKKTNGSTYQQTACSNNNSD
Shewanella         QPVSNGGSGGDNGGNDGSIEPNTQYNLVAKHSNKCIDVINSQTYNGSQYQQASCTNTNA.
Pseudoalteromonas  KPVNASNTNSVSNG..........AQIKAKHSQKCIDITAGAMSNGSYYQQWGCGSDNAN
Colwellia          KPVSNNS.....NN..TSTIPTGLTTLKAKHSNKCLDTK.GSMINGGEYHQWGCNINNAN
Catenovulum        KPVAT.......GG..GVTVPGGFNNLQAKHSNKCIDVANGAMTNGTTYHQWGCSTANVN
Gayadomonas        KPVAT..............VPSGFTNLQAKHSGKCIDLTNGAMTNGSQYQQWNCSTSNSN
Alteromonas        KPVDG..............VVTGNTNLQAKHSGRCIDVAQGAMINGSQYQQWTCDTTNTN
Aliagarivorans     KPVAEDGGGSGGDG..SVDVPSGNTNLQLVHSNLCLDVANGATWNGSTYHQWVCNTGNNN
Agarivorans        KPVTDDSSG.GENG..NVSVPSTYTNLQLVHSDLCLDVASGATWNGSTYQQWICNTGNSN
Agarivorans        KPTGGSTT..PPTG..GITPPSGYTNLQVAHSNLCLDVKSGALWNGSTYQQWSCNTGNNN
Vibrio             KPVRDND.....NN..GVVPPSDTTSLQFIHSNRCLDVKDGATWNGSTYQQWSCNTSNSN
Vas1_1339          KPVGGDD.....NG..NVTVPSGYTNTQFVHSDLCLDVKDGATWNGSTYQQWVCNTGNPN
Vibrio             KPVGGDD.....NG..DVVVPSGYTNTQFVHSDLCLDVKDGATWNGSTYQQWVCNTSNAN

1o4z
1o4z               ............................................................
Agaribacterium     QAFKFTSVGGGYYTIKSRVSNLCMDLSAGSSA..NGAKVQQWSCNSSNQNQHWKIVDKGD
Shewanella         QQFSFTELSNGFYSIKAKVSNLCLDLSAGSTA..NGAKIQQWVCNSSNQNQMWQLIDKGN
Shewanella         QKFTLNQLSNGFYTIKSNVSNLCLDLSAGSTS..NGAKIQQWVCNSSNQNQMWNLADKGD
Pseudoalteromonas  QQFNLVELSNNEYAISSQLSGLCMQIENASTS..NGAKLEQWVCDHAKASQRFTLNSTGD
Colwellia          QQFTFTHLGNSEYAITSNKSGLCVELKDANTA..NGATVHQWVCNHSENKQRWKLADKGD
Catenovulum        QGFKLVNRGNNEYTIQSERSGLCLELANGSTA..NGATIHQWVCNAANNNQRWTLHDKGD
Gayadomonas        QRFKFIALGNNEYQIQSQQSNLCLELKDGNSA..DGAVIHQWVCNSANNNQRWTLQDKGD
Alteromonas        QSFKFISAGNNEYLIQSTQSNLCVELKDNNSA..NGANIHQWVCNSANDNQKWTLHDKGD
Aliagarivorans     QRFTLSSLGNGEYALQSKVSQLCMELKDGSSS..NGATVQQWVCNHSDSNQRWSLVDKGS
Agarivorans        QRFQFSALGNGQYAISSEVSQLCLELDQASHA..DGATVHQWVCNHSDSKQTWTLFDKGS
Agarivorans        QRFQFTALGNNEYSISAKVSQLCMELASGSSA..DGAKVQQWVCNHANTSQVWSLVDKGS
Vibrio             QRFTFTPVSNSEYLIQSDNSQLCVELKPDASQWKDGATIQQYICNSAEENQLWTLFDKGS
Vas1_1339          QRFGFEEVATGEYLIQSKRSQLCLELKPGSNQWQDGAIIQQYVCNSAEENQRWTLFDKGS
Vibrio             QRFEFDEVSSGEYLIKSKRSQLCLELKPGNNQWQDGTVIQQYVCNSAEENQRWTLFDQGS

1o4z
1o4z               ............................................
Agaribacterium     AWFELRSRKANKCLDVAGKSTKNGADYVHWACYNGHNQQFRFH.
Shewanella         DYYEIRNKTSNKCIDVAGKLTTNGANITQWSCYDGDNQRFKLN.
Shewanella         GYYEIISKVSNKCLDIAGKSTNDGASLTQWSCYNGTNQQFKVE.
Pseudoalteromonas  GYFELKSSLSNKCVDIAGKLQTDGADIVQWQCYNGDNQRFQFIE
Colwellia          GYYEIRSKVSNKCLDVAGQQTNNGAAFNQWNCFNGNNQRFTFIN
Catenovulum        DYFEIHNKVSGKCVDVAGQSTDNGANIVQWNCFNGNNQRFKFLQ
Gayadomonas        QHFEIRNKITGKCIDVAGKSTTNGANIVQWSCYNGQNQRFKFLQ
Alteromonas        QHFEIRSKVTGKCIDVAGKATTNGANIVQWSCYNGQNQRFKFLQ
Aliagarivorans     NTFEIRNKASGKCLDIAGASMSNGGKLQQWACTGANNQRFRFVQ
Agarivorans        STFEIRNKVSGKCLEIANASSNNGAPLQQWSCDGGNNQRFKFL.
Agarivorans        NTFEIRNKQSGKCLEIANNSGANGADLRQWSCDGGTNQRFKFQ.
Vibrio             NTFELRNKKTGKCLEVANSQATNGGSIIQASCDGENNQRIKFK.
Vas1_1339          QTFEIRSKATGKCLELADNQGSNGGTVQQWSCDGGNNQRLKFIQ
Vibrio             QTFEIRNKATGKCLELADNQGSNGGTVQQWSCDGGNNQRLKFIQ
```
